# Intranasal Delivery of A Novel Amnion Cell Secretome Prevents Neuronal Damage and Preserves Function In A Mouse Multiple Sclerosis Model

**DOI:** 10.1038/srep41768

**Published:** 2017-01-31

**Authors:** Reas S. Khan, Kimberly Dine, Bailey Bauman, Michael Lorentsen, Lisa Lin, Helayna Brown, Leah R. Hanson, Aleta L. Svitak, Howard Wessel, Larry Brown, Kenneth S. Shindler

**Affiliations:** 1 Scheie Eye Institute and FM Kirby Center for Molecular Ophthalmology, University of Pennsylvania, Philadelphia, Pennsylvania, USA; 2 School of Veterinary Medicine, University of Pennsylvania, Philadelphia, Pennsylvania, USA; 3 Drexel University School of Medicine, Philadelphia, Pennsylvania, USA; 4 Perelman School of Medicine, University of Pennsylvania, Philadelphia, Pennsylvania, USA; 5 HealthPartners Institute, St. Paul, Minnesota, USA; 6 Noveome Biotherapeutics, Inc., Pittsburgh, Pennsylvania, USA.

## Abstract

The ability of a novel intranasally delivered amnion cell derived biologic to suppress inflammation, prevent neuronal damage and preserve neurologic function in the experimental autoimmune encephalomyelitis animal model of multiple sclerosis was assessed. Currently, there are no existing optic nerve treatment methods for disease or trauma that result in permanent vision loss. Demyelinating optic nerve inflammation, termed optic neuritis, induces permanent visual dysfunction due to retinal ganglion cell damage in multiple sclerosis and experimental autoimmune encephalomyelitis. ST266, the biological secretome of Amnion-derived Multipotent Progenitor cells, contains multiple anti-inflammatory cytokines and growth factors. Intranasally administered ST266 accumulated in rodent eyes and optic nerves, attenuated visual dysfunction, and prevented retinal ganglion cell loss in experimental optic neuritis, with reduced inflammation and demyelination. Additionally, ST266 reduced retinal ganglion cell death *in vitro*. Neuroprotective effects involved oxidative stress reduction, SIRT1-mediated mitochondrial function promotion, and pAKT signaling. Intranasal delivery of neuroprotective ST266 is a potential novel, noninvasive therapeutic modality for the eyes, optic nerves and brain. The unique combination of biologic molecules in ST266 provides an innovative approach with broad implications for suppressing inflammation in autoimmune diseases, and for preventing neuronal damage in acute neuronal injury and chronic neurodegenerative diseases such as multiple sclerosis.

Multiple sclerosis is a leading cause of neurologic disability in young adults, affecting over 2 million people worldwide^1^. Several immunomodulatory therapies that share an ability to reduce inflammatory episodes have been approved for treatment of multiple sclerosis. However, current medications have limited ability to prevent axonal loss and accumulation of neurologic disability. Optic neuritis, an inflammatory demyelinating disease of the optic nerve^2^, is the most common presenting sign of multiple sclerosis^3^ and involves a spectrum of visual changes ranging from mild to complete visual loss. Although optic neuritis often resolves with limited visual loss, up to 60% of patients develop permanent visual deficits^4^ that correlate with retinal ganglion cell (RGC) axonal loss, seen clinically as thinning of the retinal nerve fiber layer^5^,^6^,^7^,^8^,^9^. These studies suggest that therapies preventing permanent neuronal damage have the potential to prevent long-term visual loss following optic neuritis. The demyelinating process observed in optic neuritis has similar radiographic, histological, and biochemical appearances as other lesions throughout the central nervous system (CNS) white matter in multiple sclerosis patients. Therefore, identifying ways to reduce permanent visual deficits from optic neuritis may also lead to better interventions for preventing neurological deficits in multiple sclerosis.

The neurological repair potential of amnion epithelial cells has been shown to reduce fetal brain injury in response to intrauterine inflammation^10^. Amnion epithelial cells also modulate immune responses and suppress the inflammatory response in the EAE model of multiple sclerosis^11^. Amnion-derived Multipotent Progenitor (AMP) cells are a subpopulation of amnion epithelial cells that are grown in serum free media^12^. AMP cell wound healing therapy has been shown to reduce the incidence of laparotomy wound failure^13^. In a model of traumatic brain injury, AMP cell treatment was shown to significantly attenuate axonal degeneration and improve motor impairment^12^. However, administration of cells to treat neurological diseases and disorders of the brain is complex, possessing inherent regulatory hurdles, is supply restricted and is generally precluded by limitations of delivery routes.

In addition to cells, the secreted products of various cell types have been suggested as a means of treating inflammatory and neuronal diseases^14^,^15^. For example, AMP cells highly express b-III tubulin, a neuronal marker, and can be directed towards ectodermal-neuronal lineage when differentiated. Partially differentiated AMP cells produce neuronal-type cytokines including FGF2, GDF15, GDNF, Notch2, NRCAM, NRG1 (unpublished data), and TGFβ^16^. AMP cells also produce important growth factors and cytokines involved in tissue regeneration and wound repair including angiogenin, platelet derived growth factor BB, vascular endothelial growth factor, transforming growth factor–beta 2, amphiregulin, decorin, secreted protein, acidic and rich in cysteine, and hyaluronic acid. Anti-inflammatory factors include macrophage inhibitory cytokine-1, macrophage migration inhibitory factor, the protease dipeptidyl peptidase-IV, and the lipid resolvin D1, as well as anti-apoptotic factors such as soluble tumor necrosis factor receptor 1, soluble tumor necrosis factor-related apoptosis-inducing ligand receptor-3, Axl, and tissue inhibitors of metalloproteinases^17^. The AMP cell secretome called ST266 (formerly Amnion-derived Cellular Cytokine Solution^18^; ACCS) has been shown to enhance wound healing^19^, promote macrophage activity^20^, and exhibit both anti-inflammatory and neuroprotective properties in the treatment of a penetrating ballistic brain injury model^21^,^22^. ST266 also stimulates Schwann cell proliferation and protects neural cells from staurosporine induced apoptosis *in vitro*. Individual proteins in ST266 are found at concentrations in the pg/mL to ng/mL range^18^, with the total concentration of secretome proteins approximating 100 μg/mL. Many of these cytokines and growth factors are involved in the mechanisms proposed to explain fetal scarless wound healing^23^,^24^. Interestingly, a recent study showed that the secretome from mesenchymal stem cells promotes neuroprotection of RGCs in *ex vivo* retinal explants^25^, suggesting that the combination of factors secreted by stem cell populations may indeed prevent neuronal damage specifically to RGCs. However, there are few reports available on the neuroprotective potential of stem cell secretomes *in vivo*. Studies establishing efficient methods to deliver this type of biologic therapy to the retina and optic nerve, and demonstration of neuroprotective effects in relevant disease models are needed. In the current studies, the potential ability of the amnion cell derived ST266 secretome to reduce inflammation and prevent neuronal loss was examined in the experimental autoimmune encephalomyelitis (EAE) model of multiple sclerosis.

## Materials and Methods

### Animals

Six week old female C57/Bl6 mice were purchased from the Jackson Laboratory (Bar Harbor, ME, USA). Mice were housed at the animal facility at the University of Pennsylvania. All procedures were approved by and conformed to Institutional Animal Care and Use Committee at the University of Pennsylvania guidelines. Adult Sprague Dawley rats were purchased from Harlan Sprague Dawley, Inc. Rats were housed at the animal facility at the HealthPartners Institute. All procedures were approved by and conformed to Institutional Animal Care and Use Committee at the HealthPartners Institute guidelines. All applicable international, national, and institutional guidelines for the care and use of animals were followed.

### Measurement of ST266 Distribution

ST266 (Human Serum Albumin free) was radiolabeled with I-125 using the Chloramine-T (p-toluene sulfonochloramide) method^26^. Intranasal administration was performed with anesthetized Sprague Dawley rats (n = 4, 346 ± 13 grams body weight) lying on their backs. Descending aortas were cannulated for collection of blood samples during dosing and to facilitate subsequent perfusion. A pipette (P20) was used to intranasally administer 60 μl of ST266 over 18 min. Ten 6 μl nose drops were given noninvasively to alternating nares every 2 min while occluding the opposite naris. The drop was placed at the opening allowing the animal to snort the drop into the nasal cavity. 30 minutes after the onset of intranasal administration, rats were perfused with 60 ml of saline followed by 360 ml of 4% paraformaldehyde at a rate of 15 ml/min. Tissues samples were dissected and weighed, then radioactivity was assessed by gamma counting (Beckman Coulter, Indianapolis, IN, USA) and then converted into concentration units utilizing the specific activity of the administered solution and the sample weight. To assess the size of radiolabeled proteins detected in tissues, elected vitreous humor and optic nerve samples were homogenized, separated by NuPAGE^®^ Novex 10% Bis−Tris SDS gel (Thermo Fisher Scientific, Waltham, MA, USA), and analyzed for I-125 by gamma counting as in prior studies^27^.

### Induction and Scoring of EAE

EAE was induced as in prior studies^28^. Briefly, at 8 weeks of age, mice were anesthetized with isoflurane and were injected subcutaneously at two sites on the back with a total of 200 μg of myelin oligodendrocyte glycoprotein (MOG) peptide (MOG_35–55_; Genscript, Piscataway, NJ, USA) emulsified in Complete Freund’s Adjuvant (Difco, Detroit, MI, USA) containing 2.5 mg/ml mycobacterium tuberculosis (Difco). Control mice were injected with an equal volume of phosphate buffered saline (PBS) and Complete Freund’s Adjuvant. In addition, each animal received 200 ng pertussis toxin (List Biological, Campbell, CA, USA) in 0.1 ml PBS by i.p. injection at 0 h and 48 h post-immunization. Severity of EAE was scored using a previously published^28^,^29^,^30^,^31^,^32^ 5-point scale: no disease = 0; partial tail paralysis = 0.5; tail paralysis or waddling gait = 1.0; partial tail paralysis and waddling gait = 1.5; tail paralysis and waddling gait = 2.0; partial limb paralysis = 2.5; paralysis of one limb = 3.0; paralysis of one limb and partial paralysis of another = 3.5; paralysis of two limbs = 4.0; moribund state = 4.5; death = 5.0.

### ST266 Treatment

Aliquot samples of ST266 were shipped on dry ice from Noveome Biotherapeutics, Inc., Pittsburgh, PA, and stored at −20 °C. A fresh aliquot was thawed and warmed to room temperature each day to use for treatment. Mice were grouped and treated once daily with one drop (6 μl) of ST266 or PBS intranasally beginning before onset of optic neuritis (day 1 to day 42) or after (day 15–30, 15–42, 22–42 and 30–42) as indicated in each experiment. For mice receiving ST266 for only a portion of the study, the mice were treated with PBS during the interval when they were not receiving ST266 such that all control and treated mice received the same total volume of intranasal fluid daily.

### Quantification of Retinal Ganglion Cell Survival *in vivo*

RGCs were immunolabeled and counted as described previously^32^. Briefly, retinas were isolated following sacrifice, prepared as flattened wholemounts, washed and permeabilized in 0.5% TritonX 100 in PBS by freezing them for 15 min at −70 °C. The specimens were then rinsed in PBS containing 0.5% Triton, and incubated overnight at 4 °C with goat-anti-Brn3a (RGC marker) antibody (Santa Cruz) diluted 1:100 in blocking buffer (PBS, 2% bovine serum albumin, 2% Triton X 100). The retinas were washed three times in PBS, incubated for 2 hours at room temperature with anti-goat secondary antibody diluted 1:500 in blocking buffer, washed in PBS and mounted vitreous side up on slides in anti-fading solution. RGCs were photographed at 40X magnification in 12 standard fields: 1/6, 3/6, and 5/6 of the retinal radius from the center of the retina in each quadrant, and counted by a masked investigator using image analysis software (Image-Pro Plus 5.0; Media Cybernetics, Silver Spring, MD).

### Evaluation of Optic Nerve Inflammation and Demyelination

Optic nerves were isolated, fixed in 4% paraformaldehyde, embedded in paraffin, and cut into 5 μm longitudinal sections. For histological analysis, sections were stained with H&E and examined by light microscopy. Presence of inflammatory cell infiltration in the optic nerves was quantified by immunostaining for Iba1, a marker for microglia/macrophages. Optic nerve sections were deparaffinized and rehydrated. Antigen retrieval was done by heating sections at 95 °C in Vector antigen unmasking solution (Vector Laboratories, Burlingame, CA, USA) for 15 minutes. Nonspecific binding was blocked with blocking reagent from Vectastain Elite Avidin/Biotin Complex kit (ABC; Vector Laboratories), and sections were then incubated in rabbit anti-Iba1 antibody diluted 1:200 (WAKO, Richmond, VA, USA) at 4 °C overnight. Sections were washed three times with PBS and incubated with goat biotinylated anti-rabbit secondary antibody (Invitrogen, Carlsbad, CA, USA) for 2 hours at room temperature. Avidin/Biotin Complex detection was performed using the Vectastain Elite ABC kit and DAB (diaminobenzidine) substrate kit (Vector Laboratories) according to the manufacturer’s instructions. Photographs were taken at 20X magnification of three fields/nerve (one each at the distal, central, and proximal regions of the longitudinal optic nerve section) and the number of Iba1+ cells per optic nerve was counted by a blinded investigator. To detect demyelination, sections of the optic nerve were stained with Luxol fast blue (LFB) and quantified on a 0–3 point relative scale by a blinded investigator similar to prior evaluations of spinal cord demyelination^32^: 0 = no demyelination; 1 = scattered foci of demyelination; 2 = prominent foci of demyelination; and 3 = large (confluent) areas of demyelination. The entire length of each optic nerve section was examined.

### Evaluation of Spinal Cord Inflammation and Demyelination

Inflammation^30^ and demyelination^32^ in the spinal cord were assessed by previously published methods^30^,^32^. Following transcardial perfusion, spinal cords were isolated, post-fixed in 4% paraformaldehyde, embedded in paraffin, and cut into 5 μm sections. Inflammation was assessed on routine histological analysis of sections stained with hematoxylin and eosin (H&E) and examined by light microscopy. The presence or absence of inflammatory cell infiltrates, and relative degree of inflammation, was scored by a blinded investigator using a 0–3 point scale: no inflammation = 0; mild inflammation = 1, moderate inflammation = 2, severe inflammation = 3. Three sections were examined from each of three spinal cord levels (cervical, thoracic and lumbar) for each mouse. Scoring of the relative level of inflammation was based on review of all 9 sections from each spinal cord. To assess demyelination, spinal cord sections were stained with LFB. Areas of demyelination were quantified by a blinded investigator using a 0–3 point scale: 0 = no demyelination; 1 = rare foci of demyelination; 2 = a few foci of demyelination; and 3 = large (confluent) areas of demyelination. Three sections were examined from each of three spinal cord levels (cervical, thoracic and lumbar) for each mouse.

### Quantification of Axonal Area

Neurofilament staining was done according to a previously published protocol^33^. Briefly, sections of the optic nerve were deparaffinized, rehydrated, and then permeabilized with 0.5% tween-20 in PBS. Non-specific binding was blocked with blocking reagent from Vectastain Elite ABC kit (Vector Laboratories) and sections were incubated in rabbit anti-neurofilament antibody 1:100 (AbCam) at 4 °C overnight. The sections were washed three times with PBS and incubated with goat biotinylated anti-rabbit secondary antibody (Invitrogen) for 2 hours at room temperature. The ABC detection was performed using the Vectastain Elite ABC kit and DAB (diaminobenzidine) substrate kit (Vector Laboratories) according to manufacturer’s instructions. Photographs were taken of three fields/nerve (one each at the distal, central, and proximal regions of the optic nerve) by a blinded investigator using a standard exposure, and staining was quantified by calculating the optical density using Image J software (nih.gov).

### Measurement of optokinetic responses (OKR)

Visual function was assessed by OKR using OptoMotry software and apparatus (Cerebral Mechanics Inc., Medicine Hat, AB, Canada), as in prior studies^28^. OKR function is determined by the highest spatial frequency at which mice track a 100% contrast grating projected at varying spatial frequencies, and data are reported as cycles/degree.

### MitoSOX staining

MitoSOX Red (Invitrogen) mitochondrial superoxide indicator is a live-cell permeable fluorogenic dye for selective detection of superoxide in the mitochondria. MitoSOX Red reagent is selectively targeted to the mitochondria, where it is oxidized by superoxide and exhibits red fluorescence, with increased stained shown previously in EAE optic nerves^30^,^33^. Staining was performed here by the same methods. Briefly, mice were anesthetized with ketamine/xylazine and the optic nerves were removed immediately, washed with PBS and incubated in 5 μM MitoSOX Red for 30 min at 37 °C. After incubation, nerves were washed three times with PBS and then post fixed in 4% paraformaldehyde for 2 hrs at 4 °C and mounted in OCT after three washes. 5 μM cross-sections were made, viewed by fluorescent microscopy, and photographs of 5 sections/optic nerve were taken at 20X magnification. Staining was quantified by calculating the optical density using Image J software (nih.gov).

### Western Blot analysis

Retinas or optic nerves were dissected and ultra-sonicated in ice cold RIPA buffer to obtain total protein extracts as previously described^32^,^33^. Western-blot analyses were performed using antibodies raised against PGC1α (Abcam, 1:1000), SDHβ (Abcam, 1:1000), SIRT1 (Abcam, 1:1000), phosphor-AKT Ser 473 (Millipore, Billerica, MA, 1:1000), pan-AKT (Cell Signaling, Danvers, MA, 1:1000) and phosphor-PDK1 (Cell Signaling, 1:1000). β-actin (Sigma, St. Louis, MO, 1:5000) was used as control to normalize protein levels. Proteins were separated by 10% SDS polyacrylamide gel electrophoresis, with 20 μg of protein per lane, and then transferred to nitrocellulose High bound ECL membranes (GE Healthcare Biosciences, Pittsburgh, PA). The membrane was blocked with 5% Phospho blocker Blocking reagent (Cell Biolabs, San Diego CA) or 3% non-fat milk (Bio-rad, Hercules, CA) for 1 hr at room temperature and probed with primary antibody in blocking buffer overnight at 4 °C. After being washed three times using PBST, the membranes were incubated with corresponding horseradish peroxidase-conjugated secondary antibodies at a dilution of 1:3000 for 1 hr at room temperature. After three further washes, immunocomplexes were visualized with an enhanced chemiluminescence detection kit (Thermo Scientific, Southfield, MI).

### Primary RGC cultures

Primary retinal cells were prepared and cultured as in prior studies^34^. The retina was removed from 5-day old C57BL/6 J mice and cells were dissociated in solution containing papain (Worthington, Lakewood, NJ) for 15 min at 37 °C. After digestion, the retinal tissue was washed with low concentration of ovomucoid solution followed by high concentration ovomucoid solution to deactivate papain. Tissues were then transferred into Neurobasal medium containing SATO supplement, NS21 supplement and growth factors- BDNF, CNTF and Forskolin, triturated, and passed through a 40 μM cell strainer before seeding the isolated cells onto poly-D-lysine (0.1 mg/ml, molecular weight <300,000 Da, Sigma) and laminin (20 μg/ml, Sigma) coated 96 well plates at a cell density of 1 × 10^5^ cells per mL. 24 hr later, the cells were treated with 5 μM staurosporine with or without ST266 diluted 1:20 in Neurobasal medium. 2 μM of the SIRT1 inhibitor EX527, or 7 μM of pAKT inhibitor X were added where indicated. To quantify RGC survival, wells were washed twice with PBS and fixed in methanol for 15 min at −20 °C. Fixed cells were permeabilized using 0.3 M Glycine and blocked with 10% normal donkey serum in PBS for 1 h followed by incubation with anti-Thy1 antibody (Abcam, 1:100) over night at 4 °C to label RGCs. After washing with PBS, fluorescein-labeled secondary antibodies (Thermo Scientific, 1:500) were applied for 1 h at room temperature. Finally, cells were visualized and pictures were taken using a fluorescence inverted phase contrast microscope (Eclipse TE600; Nikon, Tokyo, Japan), at 10X magnification and the Thy1-specific RGCs were counted by a masked investigator.

### Statistical Analysis

Data are expressed as means ± SEM. Differences in EAE scores and OKR responses across time were compared by ANOVA of repeated measures using GraphPad Prism 5.0 (GraphPad Software, San Diego, CA). For comparisons of inflammation, demyelination, RGC numbers, RGC axon staining and visual function at individual time points, as well as protein expression by western blotting and RGC numbers *in vitro*, data were compared using one-way ANOVA followed by Tukey’s Multiple Comparison test using GraphPad Prism 5.0. Differences were considered statistically significant at p < 0.05.

## Results

### ST266 Administered Intranasally Accumulates in Optic Nerve and Vitreous

**Figure 1:**
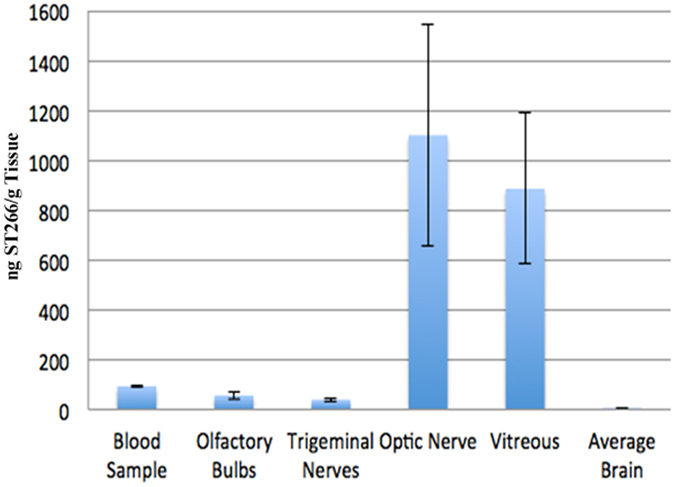
Intranasal I-125 radiolabeled ST266 accumulated at high levels in optic nerve and vitreous. 60 μl of I-125 radiolabeled ST266 was administered intranasally over 18 minutes as 6 μl drops given to alternating nares every 2 min. Animals (N = 4 Sprague Dawley rats) were euthanized 30 minutes later, tissues were removed, and levels of I-125 were measured. Significant accumulation of I-125 was detected in optic nerves (1100 ng/g of tissue) and vitreous (885 ng/g of tissue). Some radiolabeled ST266 material was also detectable in the blood, olfactory bulbs, trigeminal nerves, and across the brain, all at lower levels, less than 100 ng/g of tissue. Data represent the mean ± SD concentration measured as ng/g of tissue.

The anti-inflammatory and neuroprotective effects of intracranially delivered ST266 in a rodent model of traumatic brain injury^21^,^22^ suggested that ST266 has neuroprotective properties that could be useful to treat multiple sclerosis. However, a less invasive method of delivery for chronic disease is needed. Intranasal delivery of protein therapies has been explored as a potential therapeutic route for the CNS^35^, including delivery of the multiple sclerosis drug interferon-beta^36^. To determine if intranasal delivery could target the tissues of the eye, the biodistribution of ST266 labeled with I-125^26^ was examined in anesthetized rats. ^[I-125]^ST266 administered via the intranasal route in rats reached the CNS within 30 minutes, and was detected in the vitreous and the optic nerve at markedly higher concentrations than in the brain (Fig. 1). Approximately 0.9% of the administered dose was detected in the vitreous and about 1.1% of the administered dose was detected in the optic nerve. A high concentration in the target delivery tissues (olfactory epithelium and respiratory epithelium) as well as the olfactory bulbs (56 ng ST-266/g tissue) and trigeminal nerves (39 ng ST-266/g tissue) suggested that ^[I-125]^ST266 traveled along these pathways into the brain. In a separate experiment, SDS-PAGE chromatography of intranasally delivered radiolabeled ST266 indicated deposition of peptides/proteins in the 3000 to greater than 69,000 dalton weight range on the optic nerve and in the vitreous. These results indicated that large molecular proteins could access the optic nerve via the olfactory nerve when administered intranasally.

### Early ST266 Treatment Prevents Neuronal Damage and Dysfunction in EAE Optic Neuritis

**Figure 2:**
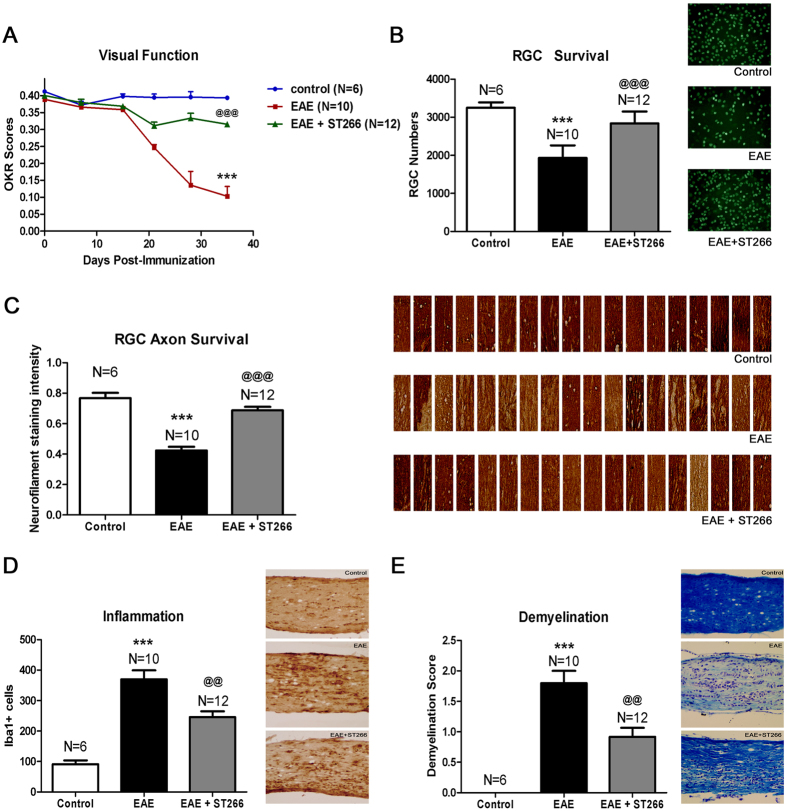
Prophylactic intranasal ST266 treatment suppresses EAE optic neuritis. EAE mice were treated with one drop (6 μl) of PBS or ST266 in the nose daily beginning on the day of immunization through the day of sacrifice 6 weeks later. Control (non-EAE) mice received daily intranasal PBS. (**A**) Visual function, measured by OKR responses, shows significant decreases in eyes of EAE mice (N = 10) compared to controls (N = 6) (***p < 0.001), and daily intranasal ST266 leads to significantly better OKR responses in EAE mice (N = 12) (^@@@^p < 0.001 vs EAE). (**B**) RGCs/retina were counted 42 days post-immunization. EAE optic neuritis induces significant RGC loss (***p < 0.001 vs. control), and ST266 prevents this loss (^@@@^p < 0.001 vs. EAE). Images show RGCs (green) in one representative retinal field from each group (original magnification X40). (**C**) Optical density of RGC axon staining in longitudinal optic nerve sections shows significant RGC axon loss in EAE (***p < 0.001 vs. control) that is attenuated by ST266 (^@@@^p < 0.001 vs. EAE). Photographs of axon staining (brown) in three regions from 6 optic nerves from each treatment group highlight the focal nature of axonal degeneration. (**D**) Macrophages/microglia in optic nerves were immunostained using anti-Iba1 antibodies. Optic nerves from EAE mice have more Iba1+ cells than control mouse optic nerves (***p < 0.001), and inflammatory cell numbers are reduced in optic nerves from ST266-treated mice (^@@^p < 0.01 vs. EAE). Images show Iba1+ cells (brown) in one representative optic nerve from each group (original magnification X20). (**E**) Myelin in optic nerves stained with LFB shows EAE mice have more myelin loss than control mouse optic nerves (***p < 0.001), and demyelination is reduced in optic nerves from mice treated with ST266 (^@@^p < 0.01 vs EAE). Images show myelin stain (blue) in one representative optic nerve from each group (original magnification X20). Data represent the mean ± SEM in all graphs.

EAE induced by immunization with MOG peptide in C57BL/6 J mice results in ascending paralysis from spinal cord inflammatory demyelinating lesions, as well as high rates of decreased visual function from optic nerve involvement in over 90% of eyes^28^,^29^. EAE was induced in 8-week old female C57BL/6 J mice to determine whether the observed levels of ST266 that accumulated in optic nerve and vitreous following intranasal administration could prevent optic nerve dysfunction and damage from optic neuritis. Mice were treated daily with a single drop (6 μl) of either ST266 (~0.6 μg of secretome proteins) or placebo (PBS) delivered intranasally beginning on the day of immunization (day 0) and continuing for 6 weeks. Prior studies^28^ have shown that RGC function decreases over time when measured by OKR, due to optic neuritis in EAE mice. Daily intranasal ST266 significantly improved OKR scores in EAE mice, with preserved levels comparable to non-EAE control mice (Fig. 2A). Intranasal ST266 significantly reduced loss of RGCs and their axons (Fig. 2B,C) in EAE mice measured 6 weeks after induction of EAE. In addition, daily intranasal ST266 treatment resulted in significant suppression of inflammatory cell infiltration of the optic nerve (Fig. 2D) and limited the degree of demyelination induced by EAE optic neuritis (Fig. 2E).

### Therapeutic ST266 Administration Reduces Neuronal Damage and Reverses Dysfunction in EAE Optic Neuritis

Initial studies detailed above (in Fig. 2) showed that ST266 treatment initiated at the time of immunization of EAE mice, before onset of optic neuritis, prevented visual dysfunction and histopathologic damage in optic nerves. Treatment at later time points was therefore examined to determine the ability of intranasal ST266 to therapeutically suppress optic neuritis after disease onset. Optic nerve inflammation begins 9–12 days post-immunization and peaks by day 15 in C57BL/6 J EAE mice, with progressive demyelination and axonal injury over the next two weeks. Significant RGC loss is detected as early as day 30, and is nearly complete by day 40^28^. EAE was induced in 8-week old female C57BL/6 J mice. Mice were treated with daily intranasal ST266, or sham treated with PBS, beginning after the peak onset of optic neuritis. All mice received one 6 μl drop of intranasal solution daily beginning on day 15 post-immunization, either ST266 or PBS as indicated. The known time course of EAE optic neuritis and the timing of ST266 treatment are shown in Supplementary Fig. 1. Table 1 summarizes the six treatment groups studied to observe the restorative effects of ST266.

**Figure 3:**
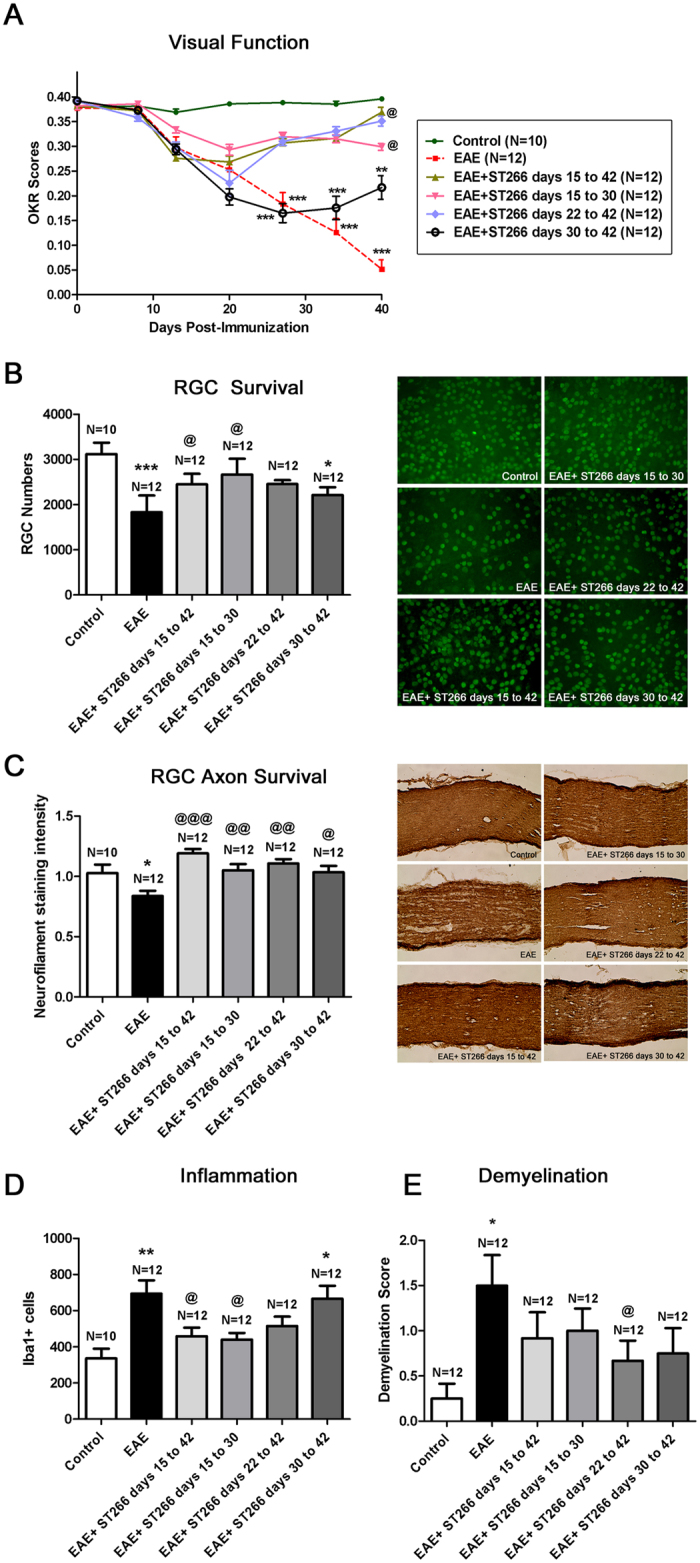
Therapeutic intranasal ST266 treatment prevents vision loss and neurodegeneration. EAE mice were treated daily with intranasal PBS or ST266 beginning 15 days post-immunization. ST266 was given on indicated days (15–42; 15–30; 22–42; or 30–42), and PBS was given all other days until euthanasia on day 42. (**A**) OKR responses show vision loss in EAE mouse eyes (***p < 0.001 vs. control). ST266 treatment days 15–30, 15–42, and 22–42 all improve OKR responses (^@^p < 0.05 vs. EAE). Despite a trend toward improvement in mice treated days 30–42, vision is reduced compared to controls (**p < 0.01). (**B**) RGC loss in EAE (***p < 0.001 vs. control) is reduced by ST266 treatment days 15–30 and 15–42 (^@^p < 0.05 vs. EAE). ST266 treatment days 22–42 and 30–42 leads to a trend in increased RGC survival, with RGC loss still occurring in eyes of mice treated from days 30–42 (*p < 0.05 vs. control). Representative images show RGCs (green) in one retina from each group (original magnification X40). (**C**) Decreased RGC axon staining in EAE (*p < 0.05 vs. control) is attenuated in all ST266 treatment groups (^@@@^p < 0.001, ^@@^p < 0.01, and ^@^p < 0.05 vs. EAE, respectively). Representative images show axons (brown) in one optic nerve from each group (original magnification X20). (**D**) Optic nerves from EAE mice have more Iba1+ macrophages/microglia than controls (**p < 0.01), and inflammation is reduced in mice treated with ST266 from days 15–30 and 15–42 (^@^p < 0.05 vs EAE). ST266 treatment days 22–42 and 30–42 leads to a trend in reduced inflammation, with significant Iba1+ cells still found in mice treated days 30–42 (*p < 0.05 vs control). (**E**) A trend toward reduction of demyelination found in EAE mice (*p < 0.05 vs. control) in ST266-treated mice was significant only in mice treated days 22–42 (^@^p < 0.05 vs. EAE). Data represent the mean ± SEM in all graphs. One representative experiment of three therapeutic treatment trials is shown.

ST266 treatment initiated at each time point halted or reversed initial RGC dysfunction after onset of treatment, marked by significantly better OKR measurements achieved at subsequent time points (Fig. 3A) as compared to EAE mice treated only with PBS. Intranasal ST266 treatment initiated at day 15 also significantly prevented RGC loss at day 42 (Fig. 3B), and treatment beginning on days 22 or 30 showed a trend toward increased RGC survival. A small but significant increase in RGC axon density was also induced by intranasal ST266 (Fig. 3C). Improved visual function and RGC survival in ST266 treated mice occurred in association with suppressed optic nerve inflammation (Fig. 3D). Reduced demyelination was also observed in ST266 treated mice compared with PBS treated mice (Fig. 3E). In contrast to effects on optic neuritis, intranasal ST266 initiated before or after onset of optic neuritis had little effect on ascending paralysis and related spinal cord inflammation and demyelination (Supplementary Fig. 2), consistent with the lower levels of ST266 that reach other parts of the CNS following intranasal delivery (Fig. 1).

### Multiple Signaling Pathways Involved in ST266 Mediated Neuroprotection

**Figure 4:**
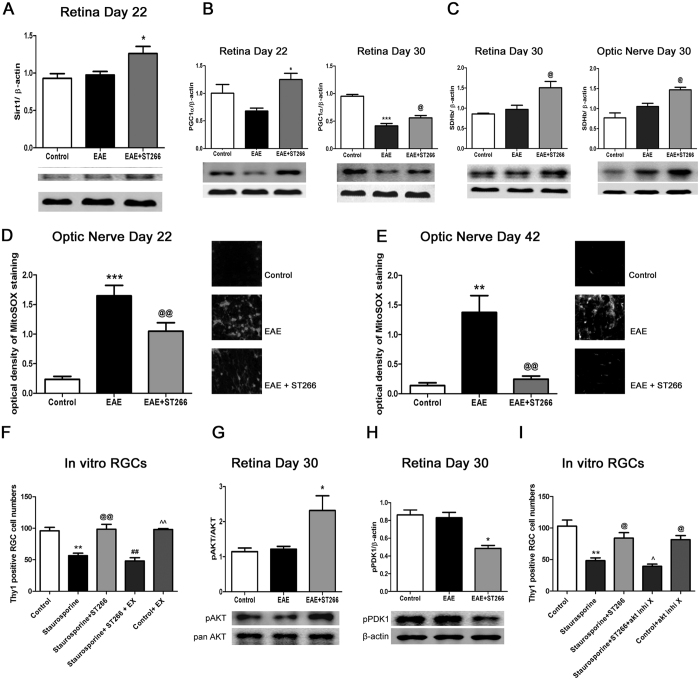
ST266-induced signaling pathways contributing to RGC neuroprotection. EAE mice were treated daily with intranasal PBS or ST266 beginning day 15 post-immunization through sacrifice (days 22, 30 or 42). Protein was isolated for Western blot analysis. Alternatively, optic nerves were stained with MitoSOX Red. Data represent mean ± SD levels from 3 mice/group. (**A**) ST266 increases day 22 retinal SIRT1 expression (*p < 0.05 vs. EAE). (**B**) ST266 increases day 22 retinal PGC1α expression (*p < 0.05 vs. EAE). By day 30, PGC1α expression in EAE retinas decreases (***p < 0.001 vs. controls), while ST266 still increases PGC1α (^@^p < 0.05 vs. EAE). (**C**) ST266 increases day 30 SDH expression in retina and optic nerves (^@^p < 0.05 vs. EAE). (**D**) Increased MitoSOX Red staining of superoxide in EAE optic nerves (***p < 0.001 vs. controls) on day 22 is reduced by ST266 (^@@^p < 0.01 vs. EAE). Images show MitoSOX Red staining in one optic nerve/group (original magnification X40) in (**D**,**E**). At day 42, increased MitoSOX Red staining in EAE optic nerves (**p < 0.01 vs controls) is further reduced by ST266 (^@@^p < 0.01 vs EAE). (**F**) Primary retinal cell cultures exposed to 5 μM staurosporine for 24 h show loss of Thy1+ RGCs (**p < 0.01 vs. controls), that is prevented by 24 h treatment with ST266 diluted 1:20 in medium (^@@^p < 0.01). 2 μM EX527 (SIRT1 inhibitor) blocks protective effects of ST266 (^##^p < 0.01). EX527 alone does not affect RGC survival (^^^^p < 0.01 vs. staurosporine +ST266 + EX527). N = 3–4 wells/culture condition. One of two experiments shown. (**G**) ST266 increases day 30 retinal pAKT expression (*p < 0.05 vs. EAE). (**H**) ST266 decreases retinal pPDK1 expression (*p < 0.05 vs. EAE). (**I**) ST266 (1:20) prevention (^@^p < 0.05) of staurosporine- induced RGC loss in primary retinal cultures (**p < 0.01 vs. controls) is blocked by 7 μM pAKT inhibitor X (^^^p < 0.05). pAKT inhibitor X alone does not affect RGC survival (^@^p < 0.05 vs staurosporine +ST266 + pAKT inhibitor X). N = 3–4 wells/culture condition. One of two experiments shown.

The numerous cytokines and growth factors present at pg to ng/mL concentrations in ST266^18^ suggest possible paracrine activation of multiple intracellular signaling pathways. Thus, ST266 itself serves as a combination therapy, but this also makes it difficult to determine all of the possible pathways involved in ST266 mediated neuroprotection of RGCs. To elucidate part of this mechanism, potential stimulation of the protein deacetylase SIRT1 was examined. SIRT1 is a NAD^+^-dependent deacetylase^37^ whose activation promotes cell stress responses and cell survival^38^,^39^. Several studies have shown that activation of SIRT1 prevents RGC loss in EAE and viral induced optic neuritis^30^,^31^,^32^,^33^, in optic nerve trauma^40^, and in primary RGC cultures^34^, by promoting mitochondrial biogenesis via deacetylation of PGC1α and resulting reduction of oxidative stress^33^,^34^,^40^. To assess the potential involvement of SIRT1 in ST266 mediated neuroprotection, EAE was induced in 8-week old female C57BL/6 J mice, and mice were treated with daily intranasal ST266, or sham treated with PBS, beginning on day 15 post-immunization. Mice were euthanized either on day 22, day 30, or day 42, and retinas and optic nerve were isolated for protein extraction or histology. ST266 treated EAE mice had increased expression of SIRT1 in the retina by day 22, as compared to PBS treated EAE mice (Fig. 4A). This was associated with increased retinal levels of the mitochondrial coenzyme PGC1α on both days 22 and 30 (Fig. 4B), and increased retinal and optic nerve levels of the mitochondrial enzyme succinate dehydrogenase, by day 30 (Fig. 4C), consistent with increased mitochondrial biogenesis in ST266 treated EAE mice. Furthermore, ST266 treatment led to a reduction in reactive oxygen species (ROS) that accumulated in the optic nerve during optic neuritis (Fig. 4D,E). RGC survival was assessed in primary retinal cell cultures to determine whether SIRT1 signaling pathways could play a functional role in preventing RGC loss. RGC death was induced by exposure to 5 μM staurosporine. Treatment with ST266 diluted 1:20 in neurobasal medium significantly attenuated RGC loss, and co-administration of 2 μM of the SIRT1 inhibitor EX527 blocked the protective effect of ST266 (Fig. 4F), suggesting that ST266 stimulated SIRT1 signaling is capable of preventing RGC death.

Multiple growth factors exert effects by stimulating the AKT signaling pathway that can reduce oxidative stress and inhibit apoptosis. For example, platelet-derived growth factor (PDGF), one of the growth factors present in ST266^18^, attenuates oxidative stress and enhances neuronal survival via PI3 kinase driven phosphorylation of AKT in primary mouse cortical neurons^41^. Thus, the potential role of phospho-AKT (pAKT) in promoting ST266 mediated RGC survival was measured. Levels of pAKT were significantly higher in retinas of EAE mice treated with intranasal ST266 beginning on day 15 post-immunization, as compared to retinas from PBS-treated EAE mice, measured on day 30 (Fig. 4G). However, phosphorylation of PDK1 (pPDK1), a target of PI3 kinase known to subsequently phosphorylate AKT^42^,^43^, was not increased. A decrease of pPDK1 was observed in the retinas of EAE mice treated with ST266 (Fig. 4H). Thus, ST266 induced phosphorylation of AKT may be mediated by another pathway, such as SIRT1, which has been shown itself to induce AKT phosphorylation^44^. Functionally, pAKT is involved in ST266 mediated RGC survival *in vitro*, as ST266 mediated neuroprotection of staurosporine-induced RGC loss in primary retinal cell cultures was blocked by 7 μM pAKT inhibitor X (Fig. 4I).

## Discussion

The current results demonstrate that intranasally delivered ST266 accumulated at therapeutic levels in optic nerve and in the vitreous, such that it reduced loss of RGCs and preserved RGC function in experimental optic neuritis. Significant decreases in inflammation and reduced demyelination of the optic nerve were also observed. Experiments suggested that the activation of SIRT1 prevented the loss of RCGs by promoting mitochondrial biogenesis and reduction of oxidative stress. The significant neuroprotective effects observed with both early ST266 administration prior to optic neuritis onset as well as later administration after disease onset suggest that ST266 represents a potential prophylactic and therapeutic treatment for optic neuritis.

Despite good recovery of visual acuity in many patients, RGC loss is associated with permanent visual dysfunction^5^,^6^,^7^,^8^,^9^ that occurs in up to 60% of optic neuritis patients^4^. More recently, studies have shown loss of RGC axons occurs in multiple sclerosis patients even in the absence of acute optic neuritis, and correlates with length of disease^45^,^46^, suggesting a progressive neurodegenerative process that is not prevented by current multiple sclerosis medications. Indeed, current therapies modulate immune responses and reduce disease relapses^47^,^48^, but show limited potential to prevent neurodegeneration and neurologic dysfunction. For optic neuritis specifically, there is no treatment that alters visual outcome. High dose corticosteroids are given to some patients to speed recovery, but this has no effect on final visual outcome^4^; and similarly, corticosteroids initiated after onset of optic neuritis do not prevent RGC loss in EAE mice^49^. Thus, improved, safe neuroprotective therapies are needed. Numerous potential neuroprotective therapies have shown promise in animal models, particularly stroke models^50^, and also in optic nerve disease^51^. However, such treatments, which typically focus on modulating one specific signaling pathway, have failed in human translational studies^50^,^51^. This failure is likely due to the complex interaction of multiple pathways in human disease, and has led to the idea that combination therapy, targeting multiple pathways is needed to provide meaningful neuroprotection.

The unique and novel composition of ST266 makes it an ideal candidate for combination therapy. The combination of biologic molecules in ST266 includes important growth factors and cytokines^16^,^18^ that can stimulate a variety of anti-inflammatory and neuroprotective responses in human cells. The near-physiologic concentrations of these factors may allow cells to respond only to those factors that stimulate receptors that have been upregulated in response to cell stress. The combination of low concentrations of cytokines and growth factors in ST266 may avoid untoward side effects and may be less likely to stimulate normal levels of receptors on healthy cells. Indeed, nine GLP preclinical acute and chronic administration safety studies of ST266 have shown it to be safe and well tolerated. ST266 has also been shown to be safe in several Phase 1 clinical trials. The multiple biologic molecules present in ST266 makes it unlikely that every specific mechanism underlying its neuroprotective effects can be fully determined, but the current studies suggest two potential signaling pathways that may be involved: AKT phosphorylation, and PGC1α deacetylation induced by SIRT1. These results are particularly intriguing based on prior studies that have shown significant RGC neuroprotective effects mediated by compounds that activate SIRT1 as well as by genetic strategies to overexpress SIRT1^30^,^31^,^32^,^33^,^34^,^40^. Of note, while prior studies show SIRT1 activators delivered either orally or intravitreally prevent RGC loss in EAE optic neuritis, the same studies found little effect on optic nerve inflammation and RGC function^30^,^31^,^32^. These results suggest that SIRT1 activators would need to be given in combination with immunomodulatory therapy to provide functional neuroprotection. ST266, however, reduced inflammation, reduced demyelination and preserved visual function in addition to preventing RGC loss whether given early or late, supporting the hypothesis that more than one pathway is likely stimulated by ST266 treatment and can combine to provide both structural and functional neuroprotection. The effects were specific on the visual pathways as shown by the fact that the reagent did not rescue spinal cord inflammation.

Current results are also consistent with prior studies showing RGC neuroprotective effects mediated by stem cells, and a recent study showing effects of a stem cell secretome on RGC survival *ex vivo*. Studies have shown that intravitreal injection of mesenchymal stem cells reduces RGC loss in models of retinal ischemia^52^, optic nerve trauma^53^, and glaucoma^54^,^55^. The mechanisms of these effects are not well understood, but are likely mediated by factors secreted by the stem cells. Johnson *et al*.^25^ recently demonstrated that the secretome of mesenchymal stem cells itself protects RGCs *ex vivo*, and identified a cocktail of several of the secreted growth factors that recapitulate these effects. One factor, PDGF, played a key role including promoting RGC survival *in vivo*. Interestingly, PDGF is one of the growth factors present in ST266^18^, and is known to signal via PI3 kinase driven phosphorylation of AKT^41^.

The potent neuroprotective results of ST266 in optic neuritis support previous studies suggesting ST266 stimulates paracrine signaling of numerous cell types to affect tissue repair^18^,^19^,^20^,^21^,^22^. Furthermore, results present the intranasal route of administration as a novel means of delivering biological protein therapeutics to the optic nerve and to the eye. Intranasal drug delivery has been used to bypass the blood-brain barrier to target the brain and CNS^35^,^36^,^56^, and clinical studies delivering insulin intranasally have been reported in Alzheimer’s disease^57^,^58^. However, specific use of any treatments for optic nerve degeneration has not been previously reported. For retinal disease, intravitreal injection has become a common delivery method for recent advancements in treatment of macular degeneration and diabetic retinopathy^59^. The potential of non-invasive intranasal administration to reach the vitreous, as seen with ST266 accumulation in vitreous in the current study, provides an opportunity for more frequent dosing with reduced risks of local complications, and would represent a huge advancement in drug delivery. Labeled proteins delivered intranasally are targeted to the cribriform plate and travel along the olfactory nerve into the CNS^60^. Rapid accumulation at the optic nerve after administration suggests localized diffusion, perhaps through lymphatic channels or along neuronal pathways more likely than systemic absorption with hematologic spread. Additional studies will be needed to determine whether similar drug accumulation occurs in humans. However, the observed distribution and activity of ST266 in rodents alone has significant implications for future drug treatment studies, as this method could potentially be used for daily intraocular dosing of drugs in place of intravitreal injections in preclinical studies. Intravitreal injections can be performed in rodent eyes^31^, but the ability to repeat injections is limited.

The current results show potent neuroprotective and anti-inflammatory effects of intranasal ST266 on the optic nerve in an animal model of multiple sclerosis. Results suggest ST266 is a potential therapy for optic neuritis, a neurodegenerative disorder of the optic nerve that currently has no available treatment to prevent RGC loss, prevent related permanent visual dysfunction, and preserve visual function. ST266 has shown an excellent safety profile in other preclinical disease models, and indeed has been and is being evaluated in several clinical trials, including in patients undergoing radiation for breast cancer (ClinicalTrials.gov Identifier: NCT01714973), for the treatment of human gingivitis (NCT02071199), periodontitis (NCT02761993) and dry eye (NCT02369861). The evaluation of drug distribution via intranasal delivery, or perhaps through intravitreal methods, may lead rapidly to translation into human clinical trials for optic neuritis. Current results further support exploration of the potential therapeutic effects of ST266 in other optic neuropathies, as well as other CNS lesions in multiple sclerosis. Intranasal ST266 represents a novel therapy and delivery route poised to fill the unmet need for neuroprotection of optic nerve diseases including glaucoma, inflammatory, ischemic and hereditary optic neuropathy, optic nerve injury and acquired and hereditary retinal degenerations. Leveraging the unique attributes of the biologic molecules present in the ST266 amnion-derived secretome may allow targeting of other neuro-degenerative targets include CNS trauma, ischemia and enhancement of anti-inflammatory treatment strategies for autoimmune diseases through innovative, non-invasive delivery methods. Furthermore, intravitreal injections are currently used to treat millions of patients with degenerative retinal diseases including macular degeneration and diabetic retinopathy. The current results suggest intranasal delivery should be explored as a potentially safer, easier and noninvasive method of delivering existing therapies that could lead to significant changes in current treatment practices.

## Additional Information

**How to cite this article**: Khan, R. S. *et al*. Intranasal Delivery of A Novel Amnion Cell Secretome Prevents Neuronal Damage and Preserves Function In A Mouse Multiple Sclerosis Model. *Sci. Rep.* **7**, 41768; doi: 10.1038/srep41768 (2017).

**Publisher's note:** Springer Nature remains neutral with regard to jurisdictional claims in published maps and institutional affiliations.

## Supplementary Material

Supplementary Figures

## Figures and Tables

**Table 1:** ST266 Treatment Groups.

Group	Treatment Cohort	ST266 (treatment days)	PBS (treatment days)
1	Control wild type mice	—	15–42
2	EAE mice	—	15–42
3	EAE mice	15–42	—
4	EAE mice	15–30	31–42
5	EAE mice	22–42	15–21
6	EAE mice	30–42	15–29

## References

[b1] Noseworthy J. H. . Multiple sclerosis. N. Engl. J. Med. 343, 938–952 (2000). 11006371 10.1056/NEJM200009283431307

[b2] Arnold A. C. Evolving management of optic neuritis and multiple sclerosis. Am. J. Ophthalmol. 139, 1101–1108 (2005). 15953446 10.1016/j.ajo.2005.01.031

[b3] Optic Neuritis Study Group. The clinical profile of optic neuritis. Arch. Ophthalmol. 109, 1673–1678 (1991). 1841573 10.1001/archopht.1991.01080120057025

[b4] Beck R. W. . A randomized, controlled trial of corticosteroids in the treatment of acute optic neuritis. N. Engl. J. Med. 326, 581–588 (1992). 1734247 10.1056/NEJM199202273260901

[b5] Steel D. H. W. & Waldock A. Measurement of the retinal nerve fibre layer with scanning laser polarimetry in patients with previous demyelinating optic neuritis. J. Neurol. Neurosurg. Psychiatry 64, 505–509 (1998). 9576544 10.1136/jnnp.64.4.505 PMC2170052

[b6] Parisi V. . Correlation between morphological and functional retinal impairment in multiple sclerosis patients. Invest. Ophthalmol. Vis. Sci. 40, 2520–2527 (1999). 10509645

[b7] Trip S. A. . Retinal nerve fiber layer axonal loss and visual dysfunction in optic neuritis. Ann. Neurol. 58, 383–391 (2005). 16075460 10.1002/ana.20575

[b8] Fisher J. B. . Relation of visual function to retinal nerve fiber layer thickness in multiple sclerosis. Ophthalmol. 113, 324–332 (2006). 10.1016/j.ophtha.2005.10.040 16406539

[b9] Costello F. . Quantifying axonal loss after optic neuritis with optical coherence tomography. Ann. Neurol. 59, 963–969 (2006). 16718705 10.1002/ana.20851

[b10] Yawno T. . Human amnion epithelial cells reduce fetal brain injury in response to intrauterine inflammation. Dev. Neurosci. 35, 272–282 (2013). 23571644 10.1159/000346683

[b11] McDonald C. A. . Immunosuppressive potential of human amnion epithelial cells in the treatment of experimental autoimmune encephalomyelitis. J. Neuroinflammation 12, 112 (2015). 26036872 10.1186/s12974-015-0322-8 PMC4457975

[b12] Chen Z. . Synergism of human amnion-derived multipotent progenitor (AMP) cells and a collagen scaffold in promoting brain wound recovery: pre-clinical studies in an experimental model of penetrating ballistic-like brain injury. Brain Res. 1368, 71–81 (2011). 20951684 10.1016/j.brainres.2010.10.028

[b13] Xing L., Franz M. G., Marcelo C. L., Smith C. A., Marshall V. S. & Robson M. C. Amnion-derived multipotent progenitor cells increase gain of incisional breaking strength and decrease incidence and severity of acute wound failure. J. Burns Wounds 7, 39–52 (2007). PMC2064968 18091982

[b14] Bernardo M. E. & Fibbe W. E. Mesenchymal stromal cells: sensors and switchers of inflammation. Cell Stem Cell 13, 392–402 (2013). 24094322 10.1016/j.stem.2013.09.006

[b15] Rosell A. . Factors secreted by endothelial progenitor cells enhance neurorepair responses after cerebral ischemia in mice. Plos One 8, e73244 (2013). 24023842 10.1371/journal.pone.0073244 PMC3762828

[b16] Banas R., Miller C., Guzik L. & Zeevi A. Amnion-derived multipotent progenitor cells inhibit blood monocyte differentiation into mature dendritic cells. Cell Transplant 23, 1111–1125 (2014). 23849060 10.3727/096368913X670165

[b17] Du Y. . Effect of Human Amnion-derived Multipotent Progenitor Cells on Hematopoietic Recovery after Total Body Irradiation in C57BL/6 Mice. Int. J. Rad. Res., in press.

[b18] Steed D. L. . Amnion-derived Cellular Cytokine Solution, A Physiological Combination of Cytokines for Wound Healing. Eplasty 8, 157–165 (2008). PMC2311453 18461121

[b19] Franz M. G.. The use of amnion-derived cellular cytokine solution to improve healing in acute and chronic wound models. Eplasty 8, e21 (2008). 18470282 PMC2323202

[b20] Uberti M. G. . Amnion-derived cellular cytokine solution promotes macrophage activity. Ann. Plast. Surg. 66, 575–580 (2011). 21451377 10.1097/SAP.0b013e318212f1d0

[b21] Deng-Bryant Y. . Treatment with amnion-derived cellular cytokine solution (ACCS) induces persistent motor improvement and ameliorates neuroinflammation in a rat model of penetrating ballistic-like brain injury. Neurol. Neurosci. 33, 189–203 (2015). 10.3233/RNN-140455 25588460

[b22] Deng-Bryant Y. . Long-term administration of amnion-derived cellular cytokine suspension promotes functional recovery in a model of penetrating ballistic-like brain injury. J. Trauma Acute Care Surg. 73, Supp 1, S156–S164 (2012). 22847087 10.1097/TA.0b013e3182625f5f

[b23] Baker R. . Cutaneous Scarring: A Clinical Review. Dermatol. Res. and Practice 2009, 1–7 (2009). 10.1155/2009/625376 PMC2879602 20585482

[b24] Mast B. A. . Scarless wound healing in the mammalian fetus. Surg. Gynecol. & Obstetrics 174, 441–450 (1992). 1570625

[b25] Johnson T. V. . Identification of retinal ganglion cell neuroprotection conferred by platelet derived growth factor through analysis of the mesenchymal stem cell secretome. Brain 137, 503–519 (2014). 24176979 10.1093/brain/awt292 PMC3914467

[b26] Hunter W. M. & Greenwood F. C. Preparation of iodine-131 labeled human growth hormone of high specific activity. Nature 194, 495–496 (1962). 14450081 10.1038/194495a0

[b27] Nguyen C. B. . Tissue Disposition And Pharmacokinetics Of Recombinant Human Nerve Growth Factor After Acute And Chronic Subcutaneous Administration In Monkeys. Drug Metabolism And Disposition 28, 598–607 (2000). 10772641

[b28] Quinn T. . Optic neuritis and retinal ganglion cell loss in a chronic murine model of multiple sclerosis. Front. Neurol. 2, 50 (2011). 21852980 10.3389/fneur.2011.00050 PMC3151613

[b29] Shindler K. S. . *In vivo* detection of experimental optic neuritis by pupillometry. Exp. Eye Res. 100, 1–6 (2012). 22561341 10.1016/j.exer.2012.04.005 PMC3923364

[b30] Shindler K. S. . Oral resveratrol reduces neuronal damage in a model of multiple sclerosis. J. Neuro-Ophthalmol. 30, 328–339 (2010). 10.1097/WNO.0b013e3181f7f833 PMC3312784 21107122

[b31] Shindler K. S. . SIRT1 activation confers neuroprotection in experimental optic neuritis. Invest. Ophthalmol. Vis. Sci. 48, 3602–3609 (2007). 17652729 10.1167/iovs.07-0131 PMC1964753

[b32] Fonseca-Kelly Z. . Resveratrol neuroprotection in a chronic mouse model of multiple sclerosis. Front. Neurol. 3, 84 (2012). 22654783 10.3389/fneur.2012.00084 PMC3359579

[b33] Khan R. S. . SIRT1 activating compounds reduce oxidative stress mediated neuronal loss in viral induced CNS demyelinating disease. Acta Neuropathol. Commun. 2, 3 (2014). 24383546 10.1186/2051-5960-2-3 PMC3892130

[b34] Khan R. S. . SIRT1 activating compounds reduce oxidative stress and prevent cell death in neuronal cells. Front. Cell. Neurosci. 6, 63 (2012). 23293585 10.3389/fncel.2012.00063 PMC3533205

[b35] Dhuria S. V., Hanson L. R. & Frey W. H. 2nd Intranasal delivery to the central nervous system: mechanisms and experimental considerations. J. Pharm. Sci. 99, 1654–1673 (2010). 19877171 10.1002/jps.21924

[b36] Thorne R. G. . Delivery of Interferon-ß to the Monkey Nervous System Following Intranasal Administration. Neuroscience 152, 785–797 (2008). 18304744 10.1016/j.neuroscience.2008.01.013

[b37] Imai S. . Transcriptional silencing and longevity protein Sir2 is an NAD-dependent histone deacetylase. Nature 403, 795–800 (2000). 10693811 10.1038/35001622

[b38] Porcu M. & Chiarugi A. The emerging therapeutic potential of sirtuin interacting drugs: from cell death to lifespan extension. Trends Pharmacol. Sci. 26, 94–103 (2005). 15681027 10.1016/j.tips.2004.12.009

[b39] Milne J. C. . Small molecule activators of SIRT1 as therapeutics for the treatment of type 2 diabetes. Nature 450, 712–716 (2007). 18046409 10.1038/nature06261 PMC2753457

[b40] Zuo L. . SIRT1 promotes RGC survival and delays loss of function following optic nerve crush. Invest. Ophthalmol. Vis. Sci. 54, 5097–5102 (2013). 23821198 10.1167/iovs.13-12157 PMC3726244

[b41] Zheng L. . Neuroprotective effects of PDGF against oxidative stress and the signaling pathway involved. J. Neurosci. Res. 88, 1273–1284 (2010). 19998489 10.1002/jnr.22302

[b42] Mora A., Komander D., van Aalten D. M. & Alessi D. R. PDK1, the master regulator of AGC kinase signal transduction. Semin. Cell Dev. Biol. 15, 161–170 (2004). 15209375 10.1016/j.semcdb.2003.12.022

[b43] Pearce L. R., Komander D. & Alessi D. R. The nuts and bolts of AGC protein kinases. Nat. Rev. Mol. Cell Biol. 11, 9–22 (2010). 20027184 10.1038/nrm2822

[b44] Pillai V. B., Sundaresan N. R. & Gupta M. P. Regulation of Akt signaling by sirtuins: its implication in cardiac hypertrophy and aging. Circ. Res. 114, 368–378 (2014). 24436432 10.1161/CIRCRESAHA.113.300536 PMC4228987

[b45] Balcer L. J., Miller D. H., Reingold S. C. & Cohen J. A. Vision and vision-related outcome measures in multiple sclerosis. Brain. 138, 11–27 (2015). 25433914 10.1093/brain/awu335 PMC4285195

[b46] Talman T. S. . Longitudinal study of vision and retinal nerve fiber layer thickness in multiple sclerosis. Ann. Neurol. 67, 749–760 (2010). 20517936 10.1002/ana.22005 PMC2901775

[b47] Frohman T. C. . Neurotherapeutic Strategies for Multiple Sclerosis. Neurol. Clin. 34, 483–523 (2016). 27445239 10.1016/j.ncl.2016.05.001

[b48] Ciccarelli O. & Thompson A. Multiple sclerosis in 2015: Managing the complexity of multiple sclerosis. Nat. Rev. Neurol. 12, 70–72 (2016). 26823149 10.1038/nrneurol.2016.2

[b49] Dutt M., Tabuena P., Ventura E., Rostami A. & Shindler K. S. Timing of corticosteroid therapy is critical to prevent retinal ganglion cell loss in experimental optic neuritis. Invest. Ophthalmol. Vis. Sci. 51, 1439–1445 (2010). 19892867 10.1167/iovs.09-4009 PMC2868414

[b50] Turner R. C. . The science of cerebral ischemia and the quest for neuroprotection: navigating past failure to future success. J. Neurosurg. 118, 1072–1085 (2013). 23331000 10.3171/2012.11.JNS12408 PMC4652647

[b51] Danesh-Meyer H. V. & Levin L. A. Neuroprotection: extrapolating from neurologic diseases to the eye. Am. J. Ophthalmol. 148, 186–191 (2009). 19464671 10.1016/j.ajo.2009.03.029

[b52] Li N., Li X. R. & Yuan J. Q. Effects of bone-marrow mesenchymal stem cells transplanted into vitreous cavity of rat injured by ischemia/reperfusion. Graefes Arch. Clin. Exp. Ophthalmol. 247, 503–514 (2009). 19084985 10.1007/s00417-008-1009-y

[b53] Zhao T., Li Y., Tang L., Li Y., Fan F. & Jiang B. Protective effects of human umbilical cord blood stem cell intravitreal transplantation against optic nerve injury in rats. Graefes Arch. Clin. Exp. Ophthalmol. 249, 1021–1028 (2011). 21360302 10.1007/s00417-011-1635-7

[b54] Yu S., Tanabe T., Dezawa M., Ishikawa H. & Yoshimura N. Effects of bone marrow stromal cell injection in an experimental glaucoma model. Biochem. Biophys. Res. Commun. 344, 1071–1079 (2006). 16643846 10.1016/j.bbrc.2006.03.231

[b55] Johnson T. V., Bull N. D., Hunt D. P., Marina N., Tomarev S. I. & Martin K. R. Neuroprotective effects of intravitreal mesenchymal stem cell transplantation in experimental glaucoma. Invest. Ophthalmol. Vis. Sci. 51, 2051–2059 (2010). 19933193 10.1167/iovs.09-4509 PMC2868400

[b56] Alcalá-Barraza S. R. . Intranasal delivery of neurotrophic factors BDNF, CNTF, EPO, and NT-4 to the CNS. J. Drug Target. 18, 179–190 (2010). 19807216 10.3109/10611860903318134 PMC3732751

[b57] Reger M. A. . Intranasal Insulin Administration Dose-Dependently Modulates Verbal Memory and Plasma Amyloid-β in Memory-Impaired Older Adults. J. Alzheimers Dis. 13, 323–331 (2008). 18430999 10.3233/jad-2008-13309 PMC2804944

[b58] Craft S. . Intranasal Insulin Therapy for Alzheimer Disease and Amnestic Mild Cognitive Impairment. Arch. Neurol. 69, 29–38 (2012). 21911655 10.1001/archneurol.2011.233 PMC3260944

[b59] Meyer C. H. . Routes for drug delivery to the eye and retina: Intravitreal injections. Dev. Ophthalmol. 55, 63–70 (2016). 26501462 10.1159/000431143

[b60] Renner D. B. . Intranasal delivery of insulin via the olfactory nerve pathway. J. Pharm. Pharmacol. 64, 1709–1714 (2012). 23146033 10.1111/j.2042-7158.2012.01555.x

