# Supplementary material for: Intranasal Delivery of A Novel Amnion Cell Secretome Prevents Neuronal Damage and Preserves Function In A Mouse Multiple Sclerosis Model
Source: Sci Rep. 2017 Jan 31;7:41768. doi: 10.1038/srep41768 (PMC5282572; doi:10.1038/srep41768)

Supplementary Information for:

Intranasal Delivery of A Novel Amnion Cell Secretome Prevents Neuronal Damage and Preserves Function In A Mouse Multiple Sclerosis Model

Authors: Reas S. Khan[1], Kimberly Dine[1], Bailey Bauman[2], Michael Lorentsen[3], Lisa Lin[4], Helayna Brown[3], Leah R. Hanson[5], Aleta L. Svitak[5], Howard Wessel[6], Larry Brown[6], *Kenneth S. Shindler[1]

Affiliations:

[1]Scheie Eye Institute and FM Kirby Center for Molecular Ophthalmology, University of Pennsylvania, Philadelphia, Pennsylvania.

[2]School of Veterinary Medicine, University of Pennsylvania, Philadelphia, Pennsylvania.

[3]Drexel University School of Medicine, Philadelphia, Pennsylvania.

[4]Perelman School of Medicine, University of Pennsylvania, Philadelphia, Pennsylvania.

[5]HealthPartners Institute, St. Paul, Minnesota.

[6]Noveome Biotherapeutics, Inc., Pittsburgh, Pennsylvania.

*To whom correspondence should be addressed:

Kenneth S. Shindler, MD, PhD
F.M. Kirby Center for Molecular Ophthalmology
Stellar-Chance Laboratories, 3rd Floor
422 Curie Blvd, Philadelphia, PA 19104, USA
Email: kenneth.shindler@uphs.upenn.edu
Phone: 215-662-8042
Fax: 215-573-7155

Supplementary Fig. 1. *EAE optic neuritis time course and therapeutic study design.* Diagram shows the known time course of pathologic changes during optic neuritis in MOG-induced EAE in C57BL/6J mice [28,29]. To study potential therapeutic effects of ST266, EAE was induced on day 0 (a), and intranasal ST266 treatment was initiated at the peak of optic nerve inflammation (b), midpoint of axon injury and demyelination (c), or onset of RGC loss (d). All mice were sacrificed after RGC loss is essentially complete (e).

Supplementary Figure 1

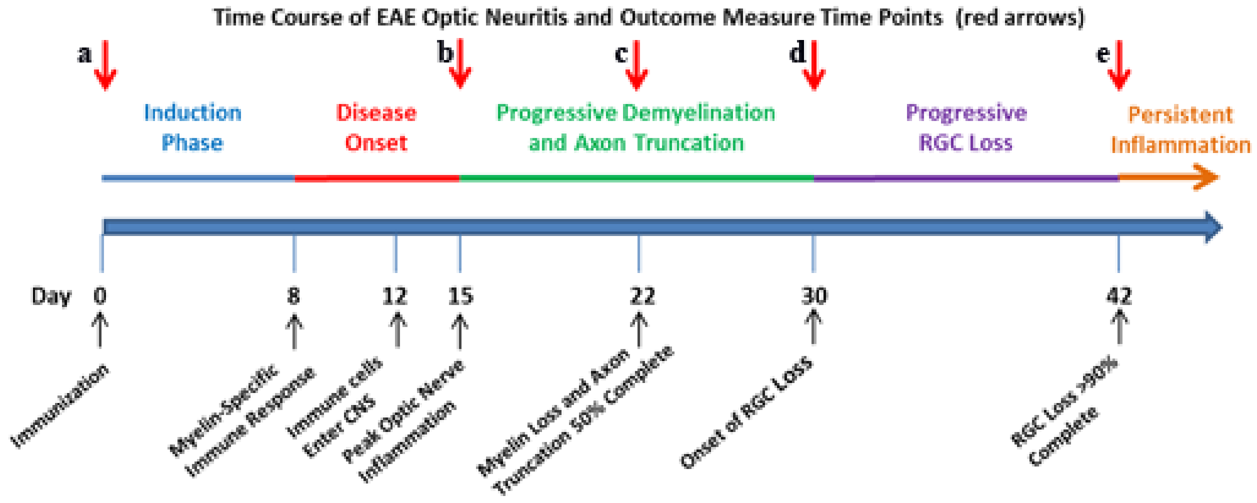

Supplementary Fig. 2. *Intranasal ST266 does not alter EAE spinal cord disease*. **A-D**. EAE mice were treated with one drop (6 μl) of PBS or ST266 in the nose daily beginning on the day of immunization through the day of sacrifice 6 weeks later. Control (non-EAE) mice received daily intranasal PBS. Clinical EAE scores, a measure of ascending spinal cord paralysis, were recorded daily. **A**. A slight trend toward decreased EAE scores in ST266-treated EAE mice (N=6) vs. PBS-treated EAE mice (N=5) was not significant. **B**. Mice were euthanized on day 42 post-immunization. Representative sections stained by H&E and LFB show normal histology and myelination in adjacent sections of one control mouse spinal cord. Examples of spinal cords from two PBS-treated and two ST266-treated EAE mice show areas of cellular infiltration and corresponding demyelinating plaques (delineated regions within dotted lines) occur in white matter in EAE mice and are not prevented by ST266 treatment. **C**. Relative inflammation levels in spinal cords of PBS-treated EAE mice are not significantly different from ST266-treated EAE mice. **D**. Relative demyelination levels in spinal cords of PBS-treated EAE mice are not significantly different from ST266-treated EAE mice. **E-G**. EAE mice (N=6/treatment group) were treated with one intranasal drop of PBS or ST266 daily beginning 15 days post-immunization. ST266 was given on indicated days (15-42; 15-30; 22-42; or 30-42) for each treatment group, and PBS was given on all other days until mice were euthanized on day 42. **E**. Relative inflammation levels in spinal cords of PBS-treated EAE mice are not significantly different from ST266-treated EAE mice. **F**. Relative demyelination levels in spinal cords of PBS-treated EAE mice are not significantly different from ST266-treated EAE mice. **G**. Variations in EAE scores between the four different ST266-treatment groups and the PBS-treated EAE mice were not statistically significant.

Supplementary Figure 2

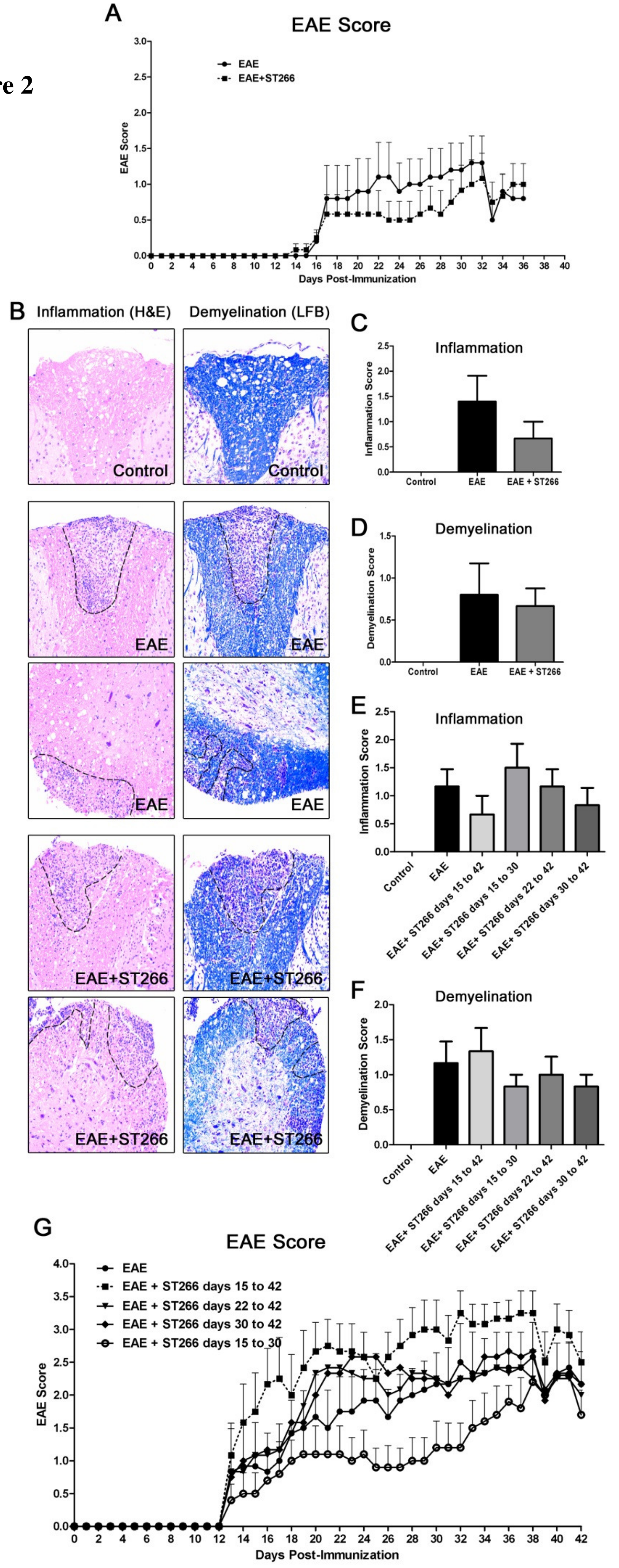

Supplement: Supplementary Figures [file srep41768-s1.pdf]
